# Denoising Two-Photon Calcium Imaging Data

**DOI:** 10.1371/journal.pone.0020490

**Published:** 2011-06-07

**Authors:** Wasim Q. Malik, James Schummers, Mriganka Sur, Emery N. Brown

**Affiliations:** 1 Department of Anesthesia, Critical Care and Pain Medicine, Massachusetts General Hospital, Harvard Medical School, Boston, Massachusetts, United States of America; 2 Department of Brain and Cognitive Sciences, Massachusetts Institute of Technology, Cambridge, Massachusetts, United States of America; 3 Picower Institute for Learning and Memory, Massachusetts Institute of Technology, Cambridge, Massachusetts, United States of America; 4 MIT/Harvard Division of Health Sciences and Technology, Massachusetts Institute of Technology, Cambridge, Massachusetts, United States of America; University of Maribor, Slovenia

## Abstract

Two-photon calcium imaging is now an important tool for *in vivo* imaging of biological systems. By enabling neuronal population imaging with subcellular resolution, this modality offers an approach for gaining a fundamental understanding of brain anatomy and physiology. Proper analysis of calcium imaging data requires denoising, that is separating the signal from complex physiological noise. To analyze two-photon brain imaging data, we present a signal plus colored noise model in which the signal is represented as harmonic regression and the correlated noise is represented as an order 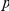 autoregressive process. We provide an efficient cyclic descent algorithm to compute approximate maximum likelihood parameter estimates by combing a weighted least-squares procedure with the Burg algorithm. We use Akaike information criterion to guide selection of the harmonic regression and the autoregressive model orders. Our flexible yet parsimonious modeling approach reliably separates stimulus-evoked fluorescence response from background activity and noise, assesses goodness of fit, and estimates confidence intervals and signal-to-noise ratio. This refined separation leads to appreciably enhanced image contrast for individual cells including clear delineation of subcellular details and network activity. The application of our approach to *in vivo* imaging data recorded in the ferret primary visual cortex demonstrates that our method yields substantially denoised signal estimates. We also provide a general Volterra series framework for deriving this and other signal plus correlated noise models for imaging. This approach to analyzing two-photon calcium imaging data may be readily adapted to other computational biology problems which apply correlated noise models.

## Introduction

Two-photon microscopy is now widely recognized as a valuable tool for real-time *in vivo* imaging of biological systems [1], [2]. A two-photon microscope excites fluorophores in a volume of biological sample using pulsed lasers to induce the emission of a fluorescence signal [3]. Typically, a focused laser beam scans the tissue in a two-dimensional raster pattern, producing a fluorescence image that typically spans hundreds of cells [4]. The images facilitate highly informative and quantitative analyses with a range of biological applications. Two-photon imaging of calcium-sensitive fluorescent indicators to investigate neural physiology is particularly appealing because the measured fluorescence is closely related to neural activity [5]. This imaging modality enables analysis of a broad spatial scale, ranging from the structure of dendritic spines (microns) [6]–[8] to the architecture of neuronal networks (millimeters) [9]–[12], as well as analysis of a broad temporal scale from fast action potentials (milliseconds) [10], [12]–[14] to slow calcium waves (seconds) [15], [16].

Properly separating signal from noise, often termed denoising, is a crucial signal processing procedure in the analysis of imaging data. While there has been considerable success in the development of two-photon microscopy hardware and experimental techniques, the corresponding signal processing methodology has received less attention. The observed fluorescence response depends upon several factors: 1) the nature of the stimulus and the modulation of neural activity due to the stimulus; 2) movements due to highly structured physiological processes; 3) spontaneous neural activity; and 4) optical and electrical noise. Current approaches to processing two-photon data consist of averaging the measured fluorescence levels over multiple trials followed by kernel-based smoothing or fitting an appropriate curve to these time-series data [11], [17]–[19]. Averaging, while highly intuitive and easy to perform, requires a large number of trials which is often not possible in two-photon imaging experiments. Principal components analysis (PCA) has also been used for denoising image data by dimension reduction [14], but in general it does not exploit the stimulus-driven modulation of the response. One PCA-based approach preserves only the PCA components that exceed a certain threshold of correlation with the stimulus sequence [20]. Fourier analysis of two-photon data recorded in response to periodic stimulation allows for signal extraction at the excitation frequency [21], [22] and possibly some of its harmonics [23], but does not model activity at other frequencies. A signal plus colored noise model has been used to analyze functional magnetic resonance (fMRI) data [24]. However, the expectation-maximization estimation algorithm used in that method has high computational complexity.

Nonlinear approaches to signal analysis could be candidates for the analysis of the data considered here. These include projective filtering, wavelet methods, local linear approximation, and several other methods [25]–[28]. We derive our approach by assuming a nonlinear relationship between the measured fluorescence response, the stimulus, and the colored noise. The Volterra series expansion leads to an approximation of this nonlinear relationship that relates the measured fluorescence linearly to the harmonic signal and the correlated noise. Our analysis shows that this approach to constructing models for two-photon imaging also yields the commonly used signal plus noise models for fMRI data analysis.

We propose a novel statistical signal plus correlated noise (SCN) model for the analysis of the two-photon calcium imaging data in which the stimulus induced structure is represented as a harmonic regression and the temporally correlated noise is represented as an autoregressive process. We present a computationally efficient cyclic descent algorithm for maximum likelihood estimation of the model parameters. Our approach differs from current two-photon image analysis techniques in that we use a formal likelihood framework to not only separate signal and noise, but to also select a model, assess model goodness of fit and make inferences about physiological features in the estimated images. The high computational efficiency of the algorithm makes it amenable to automated analysis of large imaging data sets. By analyzing two-photon calcium imaging data recorded from the ferret primary visual cortex, we demonstrate that our approach accurately models the data and provides significantly denoised images.

## Results

### Visual stimulation and two-photon image acquisition

**Figure 1:**
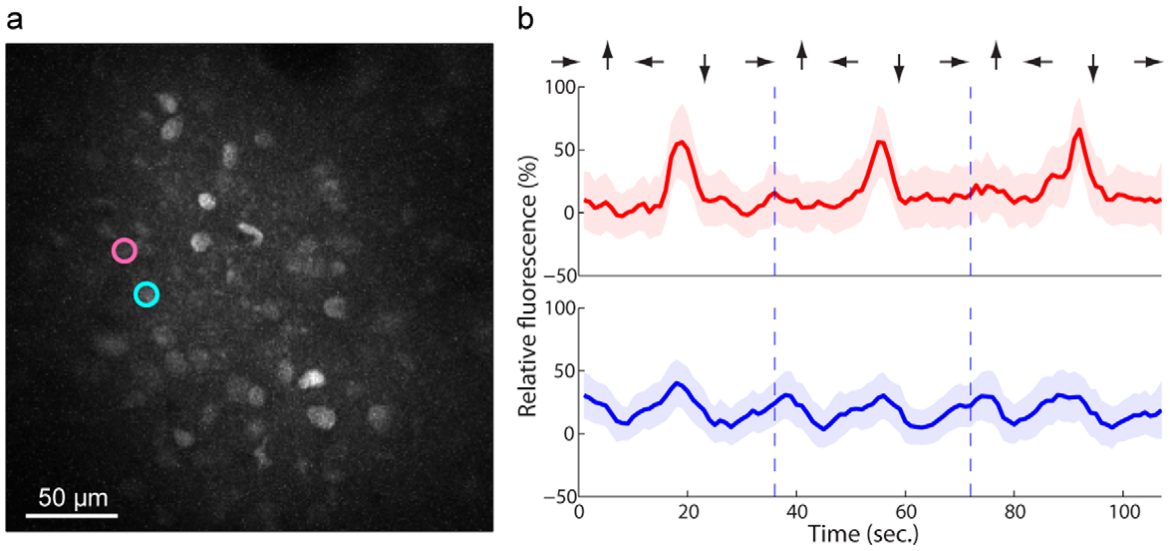
Example of two-photon fluorescence image and individual cell dynamics. (a) Anatomical image of cell population at 150 m depth obtained by plotting the pixel-wise maximum fluorescence across the movie frames obtained under a rotating orientation stimulus. Brighter shades represent higher fluorescence intensity. ROIs indicate two neighbouring neurons identified manually, Cell 11 (blue) and Cell 12 (red). (b) Relative fluorescence time-traces for each of the two neurons in three trials with stimulus orientation indicated by arrows. Solid line and background region show the mean
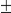
s.d. across pixels comprising the cell. Vertical dashed lines mark trial boundaries, at which the stimulus is oriented at 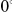 w.r.t. positive x-axis (horizontal) and then rotates counter-clockwise. The response of Cell 12, unlike Cell 11, is specific to the drift direction, exhibiting an excitatory response at horizontal orientation for one drift direction (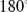 orientation) but not the other (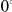 orientation).

Time series traces of two-dimensional images (XYT) with a field-of-view of approximately 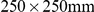 were collected at 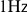 from the primary visual cortex of a ferret using a two-photon microscope (see Methods). The stimulation protocol consisted of square-wave gratings with 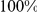 contrast which drifted at 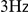 orthogonally to the orientation and rotated by 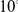 every second (each data frame), i.e., the stimulus rotated 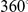 in 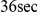. The time series of the response of a neuron to this stimulus approximated a full orientation tuning curve. This stimulus was repeated three times. Prior to recording the stimulus responses, 10 image frames were acquired in the absence of any visual stimulus and their mean provided the estimate of the baseline level at each pixel. Manually determined boundaries delineate the set of pixels that define each cell, and each of the 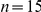 cells thus identified (Figure S1a) consists of 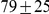 pixels (mean
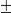
s.d.). The data consist of the time series of fluorescence on each image pixel. The relative fluorescence on a given pixel is 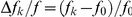, where 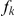 is the 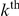 time-sample of the measured fluorescence intensity; 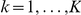; 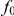 is the baseline level; and we have 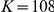 samples. Using this orientation stimulus, initial anatomical images of the neuronal population can be obtained by plotting the pixel-wise maximum fluorescence across the image time-series, 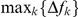 (Figure 1a). The relative fluorescence traces from the imaged cells show the diversity of orientation responses (Figure 1b and S1b).

### A signal plus correlated noise model

We assume that in each pixel, the measured fluorescence at each time can be described by a signal plus correlated noise (SCN) model defined as
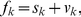
(1)


where the signal is defined as the order 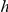 harmonic regression
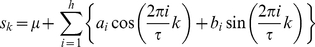
(2)


and 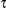 is the period of the stimulus. We assume that the temporally correlated noise obeys the 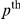 order autoregressive model (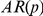) given by
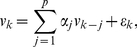
(3)


where the 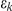 are assumed to be independent, identically distributed Gaussian random variables with mean zero and unknown variance 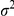. We assume that the zeros of the characteristic polynomial, 
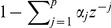
, are outside the unit circle to insure stationarity of the 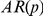 model. We model the signal as a harmonic regression because the measured fluorescence shows a strong sinusoidal response at the period of the stimulus. We postulate that this smooth, periodic structure should be well described by the low-order terms of a Fourier series expansion defined by the harmonic regression model. The 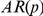 model represents the highly structured physiological and electronic noise components of the fluorescence measurements. This and other signal plus correlated noise models can be derived from the Volterra series framework that we present in Methods.

### Efficient approximate maximum likelihood parameter estimation by cyclic descent

To use the SCN model in Eq. 1 to denoise calcium imaging data, we estimate its parameters 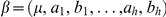, 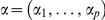 and 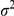 by maximum likelihood using a cyclic descent algorithm. The cyclic descent algorithm provides an efficient approach for solving this nonlinear estimation problem by iterating between computing the solutions to two highly tractable linear estimation problems (see Methods). That is, at iteration 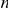, given 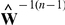 the estimate of the inverse of the covariance matrix of 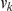 from iteration 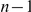, the algorithm computes 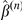, the weighted least-squares estimate of 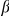. Given 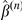, the algorithm computes 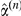 and 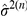 using Burg algorithm and 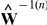 using Levinson-Durbin recursion (Methods). Because of the properties of the Burg algorithm, we provide AR parameter estimates that yield a stationary process. The Levinson-Durbin algorithm provides an efficient means of computing 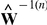 from 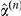 and 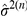. This efficiency is significant for large 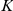 since 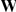 is a 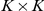 matrix. We use as the stopping criterion the condition that the relative change in the estimate of 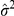 between iterations is smaller than threshold 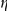. If this stopping criterion is satisfied, the algorithm stops; otherwise, given 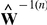, the algorithm proceeds to iteration 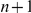. With this stopping criterion based on the residual variance, the cyclic descent algorithm applied to our calcium imaging data consistently converges in 3 to 5 iterations. This class of iterative algorithms are known to converge at least linearly [29], and our results show that the cyclic descent algorithm in fact achieves exponential convergence (Figure S2). Although this algorithm is highly efficient, we can further expedite processing, as may be required for real-time implementation, by reducing the number of iterations. This cyclic descent algorithm avoids computing the gradients and Hessians required for Newton's procedure and the multiple iterations characteristic of the expectation maximization algorithm. A theorem due to Corradi [29] suggests that our cyclic descent algorithm finds the global maximum of the likelihood.

### Choice of model order and assessment of goodness-of-fit

**Figure 2:**
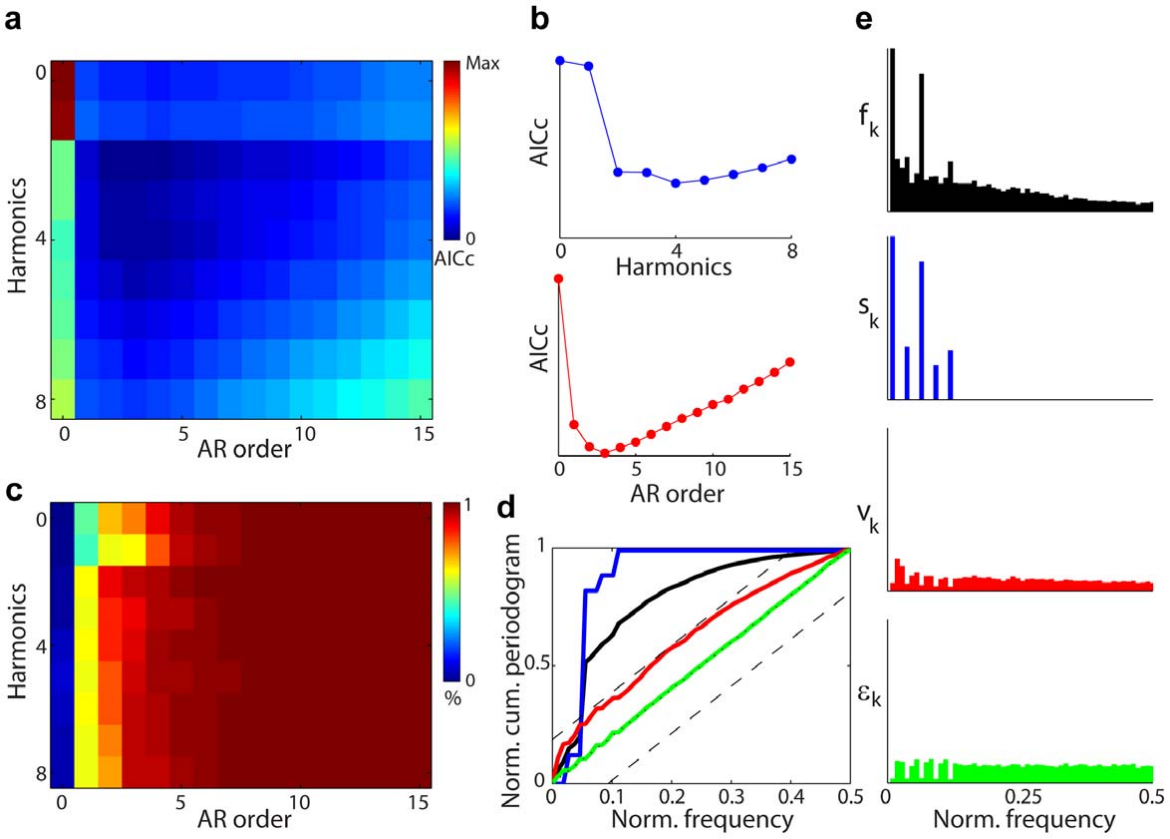
Model order selection and goodness of fit. The fluorescence time series in this example is from Cell 11 in our data set. (a) Corrected Akaike information criterion (AICc) as a function of the harmonic (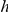) and autoregressive model orders (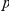). The minimum of this surface is achieved with 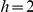 and 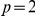. In the pseudo-color heat map, red and blue represent large and small AICc values, respectively. (b) Left: For a signal-only model (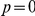), AICc suggests 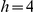 as optimal. Right: For the SCN model with 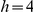, AICc suggest 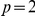 as optimal. (c) Percentage of the cell's pixels for which the SCN model yields substantially white residuals for given 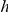 and 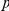. With 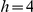, the whiteness criterion is satisfied for all pixels by 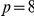. In the pseudo-color heat map, blue and red represent 0 and 100% respectively. (d) Normalized cumulative periodograms (NCPs) of the data and its component estimates (solid lines) averaged across the cell's pixels: measured fluorescence data 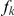 (black), stimulus-evoked signal component 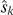 (blue), stimulus-free background activity 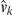 (red), and residual white noise 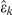 (green). Also shown are 95% whiteness bounds (dashed lines) and NCP of ideal white noise (dotted line). Temporal correlation in background activity is evident from its highly nonuniform spectrum, while the residuals lie within the whiteness bounds. (e) Spectra illustrating decomposition of a pixel's fluorescence time series data into estimate signal component, background activity, and residual white noise. Dominant lower-frequency components in the data spectrum correspond to the stimulus response and are captured by 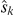 which has a line spectrum at the first 4 harmonics of the stimulus frequency. The remaining activity, with a nonuniform spectrum, is captured by the AR component. The spectrum of the residual noise is substantially uniform, confirming whiteness.

Separation of the fluorescence data into signal and correlated noise relies on choosing appropriate values of model orders 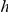 and 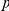. To make these selections, we use well-established model selection and goodness-of-fit criteria, namely the corrected Akaike information criterion (AICc) and analyses of the correlation structure and spectra of the residuals from the model fits (Methods). These criteria help determine the optimal tradeoff between model parsimony and estimation accuracy. As a representative example, we consider the AICc for various model orders for one cell (Figure 2a). The AR model alone can capture much of the periodicity in the data including that due to the stimulus response, but unlike the SCN model, it does not decompose the data into stimulus-driven and background components. Our approach therefore is to fit a signal-only model first and determine the optimal 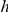 using AICc (Figure 2b). Next, using the chosen 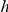, we fit the SCN model and determine the optimal 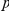 (Figure 2b). Goodness-of-fit analysis is another important consideration whose purpose is to insure that the residuals, 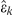, are white (Methods), confirming that the systematic variance in the data has been explained by the SCN model. We find that inclusion of an AR component is necessary to obtain white residuals even when 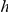 is large (Figure 2c). The AR model order suggested by AICc is insufficient and instead a higher order is required to guarantee white residuals. The fluorescence data spectrum shows certain dominant periodicities, some of which correspond to the stimulus frequency and its low harmonics, and are captured by 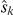 (Figure 2d). The nonuniform spectrum of background activity, including the significant activity observed at low frequencies, is captured by the AR component, 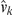. The spectrum of residuals, 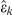, is approximately flat. The normalized cumulative periodogram (NCP) of 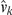, falls outside the 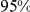 whiteness bounds (Figure 2e). In contrast, the 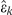 NCP nearly coincides with white noise NCP as desired. This analysis assists in determining the required harmonic and AR model orders, which may vary from cell to cell. We find that 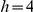 and 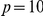 satisfy the above requirements for most of the cells in our data-set (Figure S3 and Table S1). Once the optimal SCN model order has been determined and goodness-of-fit assessment completed, we use the model to make biological inferences.

### Tuning curve estimation

**Figure 3:**
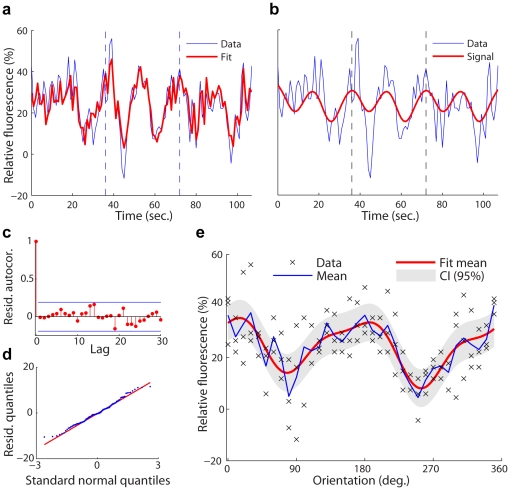
Decomposition of fluorescence data into signal and noise components. The representative time series data is from Cell 11, Pixel 45 in our data set. (a) Relative fluorescence data 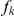 (blue) measured in three consecutive trials (dashed lines: trial boundaries). The fit (red) is the estimate 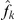 obtained by the signal plus correlated noise model, containing both stimulus-evoked activity and noise. (b) Relative fluorescence data 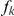 (blue) in three consecutive trials and estimate (red) of the signal component 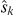 (i.e., the stimulus-evoked activity). (c) Autocorrelation function (red) of residual noise, 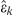, lies within the 95% whiteness bounds (blue). (d) The quantile-quantile plot of the residuals confirms Gaussianity. The results in (c) and (d) prove that the residuals are independently and identically distributed Gaussian, and the systematic variance in the data has been explained by the harmonic regression and autoregressive terms. (e) Orientation tuning curve obtained from the denoised signal estimate in (b). The SCN model provides a smooth fit to the across-trials mean of the data. Point-wise approximate 95% confidence intervals are also shown. The SCN model preserves the complex, asymmetric shape of the response tuning curve.

We use the SCN model to characterize the relative fluorescence response to the stimulus at a single pixel. The close fit between the data and the signal estimate establishes the validity of our model (Figure 3a). The signal component, 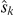, provides a denoised estimate of the response for three trials of stimulus presentation (Figure 3b). The autocorrelation function and quantiles of the residual, 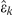, confirm that it is consistent with an independent Gaussian process (Figure 3c and d). We construct the denoised response tuning curve, 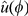, where 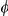 is a circular random variable that represents the stimulus orientation. We also obtain the approximate 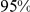 confidence intervals (Methods) and analyze the response characteristics. This signal estimate (Figure 3e) captures the key features of the neuronal response, such as the location and width of tuning to the stimulus effect. The use of a Gaussian or cosine curve to fit the data, as is common practice in neuroscience, would constrain the response estimate to have a simple, symmetric shape. Our model allows the tuning curve estimate to reflect the complex shape of the cell response observed in the data with minimal computational complexity.

### Image denoising

**Figure 4:**
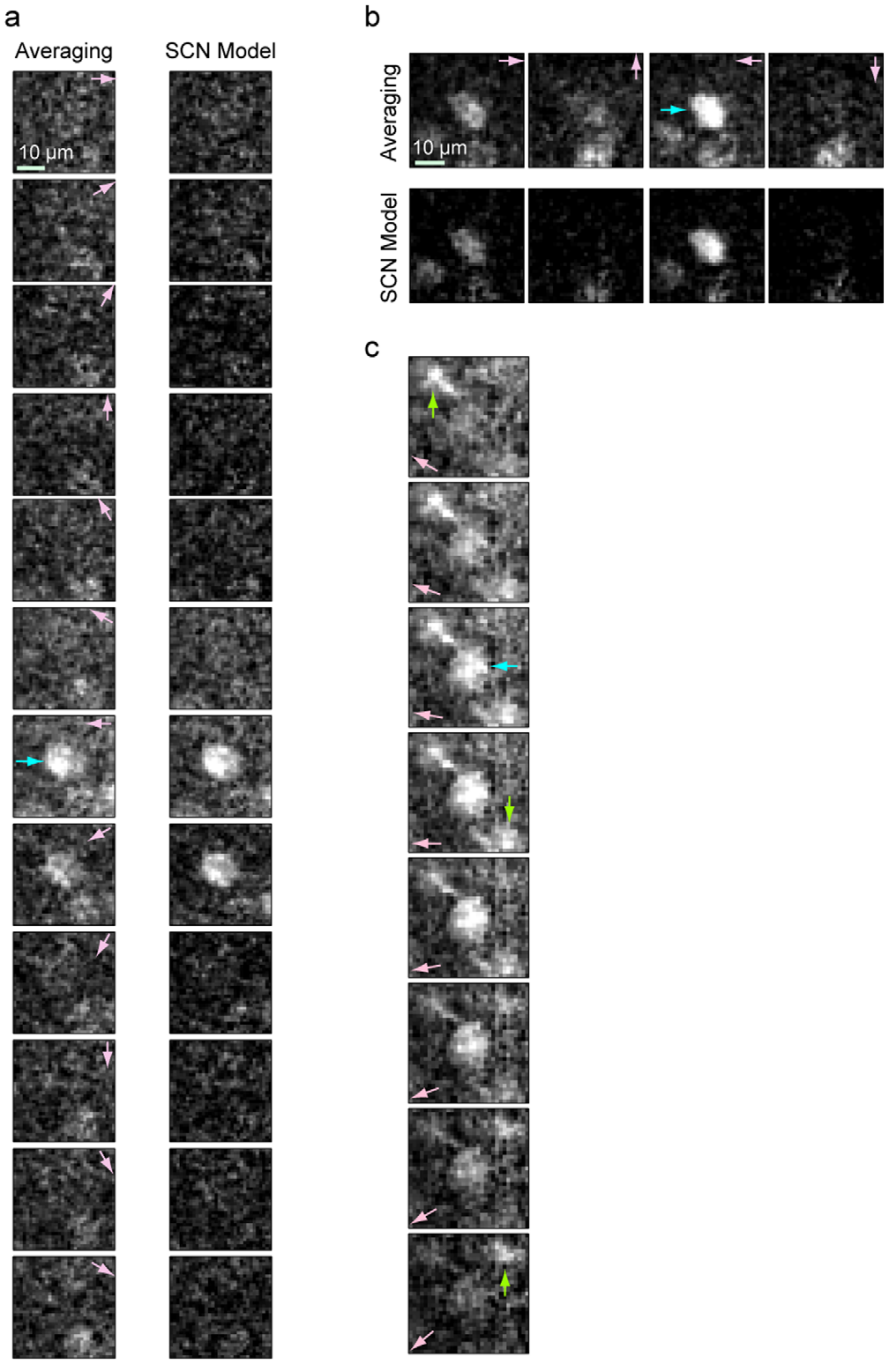
Image denoising and visualization of calcium activity. Differential fluorescence (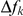) response images from the area around Cell 14 (cell soma marked by blue arrow) to orientation stimulus processed with conventional across-trial averaging and with the SCN model. Successive frames show the response at orientations (marked by pink arrows) in 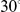 steps. The SCN model separates the background activity from the signal. (b) Fluorescence response of Cell 1 along four orientations (soma marked by blue arrow). (c) Fluorescence response of Cell 12 obtained from SCN model in 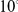 orientation steps with successive frames acquired at 1 Hz. Slow calcium waves flowing across the cell processes (green arrow) and soma (blue arrow) are observed due to the enhanced clarity of the denoised image.

We can reconstruct denoised images using the signal component estimate, 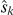, at each pixel. A comparison of the fluorescence response estimates of pixels around a cell obtained with conventional across-trial averaging and with our SCN model (Figure 4a) demonstrates the enhanced image contrast and clarity provided by our model. Our denoising method delineates the stimulus response within the cell soma and allows improved observation of calcium dynamics around the cell associated with excitation. In a second cell (Figure 4b), the background activity at the bottom of the frame is substantially reduced in intensity by our approach. The increased contrast of the denoised images reveals additional subcellular processes not discernible in the conventional images obtained by averaging (Figure 4c). This opens up the possibility of future work to characterize the source of these signals and their behavior.

### Inferring neuronal characteristics

**Figure 5:**
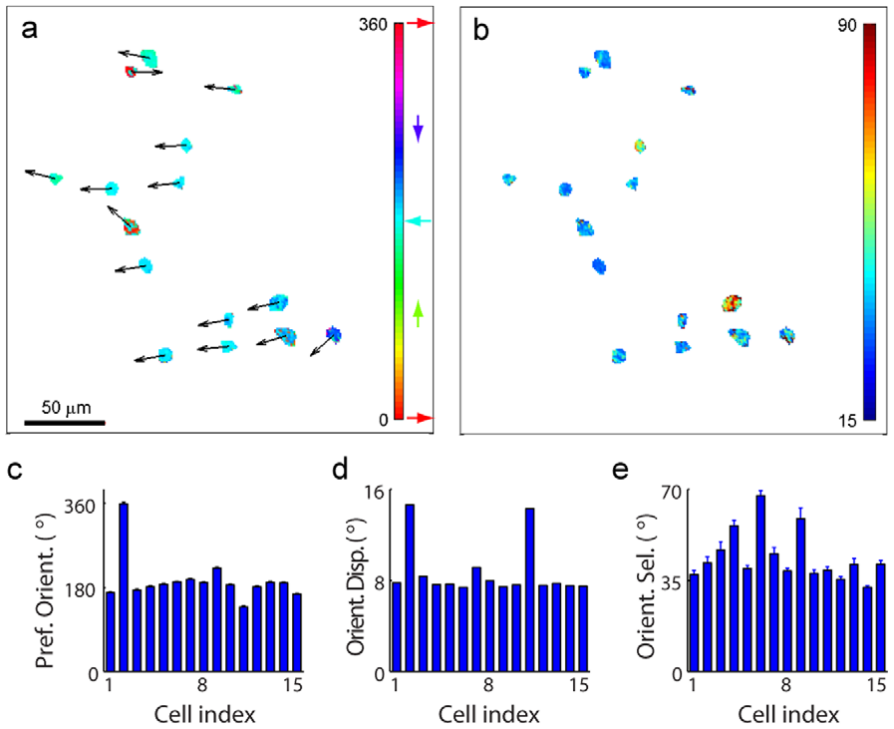
Spatial distribution of preferred orientation and orientation selectivity after denoising with SCN model. (a) Visual response characteristics for pixels within cell boundaries identified manually from Figure 1a. Pixel-wise preferred orientation is indicated by the colormap, and mean preferred orientation of each cell is indicated by the black arrow originating from the cell center. Colorbar and colored arrows represent the orientation scale in degrees. (b) Orientation selectivity, a measure of tuning sharpness evaluated as the half-width range at half-height response, is indicated by the colormap. Colorbar represents angle in degrees. (c) Cell-wise preferred orientation according to cell indices defined in Figure S1a, evaluated as the mean preferred orientation across all pixels in a cell (errorbars indicate 95% confidence intervals on the mean). (d) Circular dispersion of cellular orientation response. (e) Orientation selectivity of the cell (mean
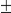
s.e.m. across pixels).

By denoising two-photon imaging data with the SCN model, we provide reliable estimates of several quantities that may be used to describe neuronal behavior. For example, we study the orientation preferences of the primary visual cortex neurons in our sample. At each pixel, the preferred orientation is obtained as the orientation at which the denoised tuning curve peak occurs, i.e., 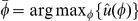. As previously reported [9], neighboring cells show a preference for similar orientations with a smooth spatial variation (Figure 5a and c). Among the cells, there are different degrees of deviation from the mean preferred orientation (Figure 5d). This deviation is particularly high for two of the cells possibly due to somatic and dendritic dynamics. We calculate the orientation selectivity from the estimated tuning curve, 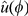, as the half-width at half-height. Our analysis of orientation selectivity at each pixel reveals both spatial trends and intra-cellular variations (Figure 5b). A wide-ranging level of orientation selectivity is apparent (Figure 5e). These examples demonstrate that the SCN model can facilitate a variety of functional analyses with high reliability.

### Neuronal signal-to-noise ratio estimation

The ratio of stimulus-evoked response (signal) to background activity (colored noise) provides a natural definition of the neuronal signal-to-noise ratio (SNR) and a way to compare the relative responsiveness of the cells to the stimulus. We calculate the signal power, 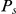 from our harmonic model and the noise power, 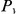, from the AR model to obtain
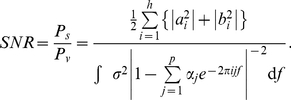
(4)


**Figure 6:**
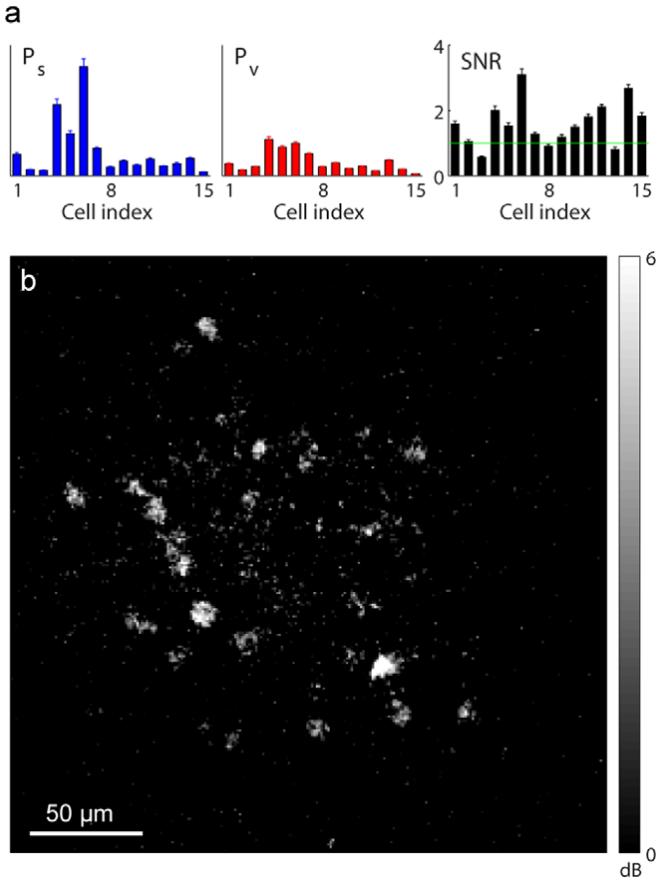
Neuronal signal-to-noise ratio (SNR) estimated using the SCN model. The SNR is the ratio of the power of stimulus-evoked signal to that of stimulus-free background activity. (a) Signal power (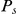), noise power (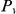), and SNR of all 15 neurons (mean
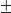
s.e.m. computed across pixels). (b) Spatial map of SNR at each pixel (shown in decibel scale) agrees closely with the anatomical map in Figure 1a.

The cells in our data set exhibit a wide range of SNRs (Figure 6a). The locations of the cells with high SNR (Figure 6b) agree closely with the anatomical map (Figure 1a), and therefore the pixel-wise SNR maps can be used to identify robustly the locations of cells that respond to the given stimulation.

## Discussion

We have presented a flexible local likelihood framework for analyzing two-photon calcium imaging data. Our framework appreciably enhances image contrast on a pixel-by-pixel basis by using an SCN model to separate the salient stimulus-evoked neural responses from the complex forms of physiological and recording noise common to two-photon imaging experiments. The cyclic descent algorithm provides a computationally efficient approach for fitting the SCN model to the time-series of fluorescence responses. Our framework suggests a straight-forward yet novel way to track with improved subcellular resolution the temporal dynamics of individual neurons (Figure 4) and obtain significantly denoised images of neuronal populations (Figure 5).

We have formulated our analysis as a harmonic regression plus colored noise problem. Cellular calcium responses have a stochastic nature and exhibit oscillations with a colored noise component [30], as demonstrated by calcium recordings from pancreatic acinar cells [31] and airway myocites [32]. Colored noise also appears in many other contexts in computational biology, including functional magnetic resonance imaging (fMRI) [24], [33], neural voltage-sensitive dye imaging [34], circadian rhythms [35], synaptic background activity in cortical neurons [36]–[38], gene regulatory networks [39], speech signals [40], cell locomotion patterns [41], and many others. The procedures usually applied to these problems, based on expectation maximization or exact maximum-likelihood procedures, are often computationally intensive. Our approach suggests an alternate approximate maximum likelihood procedure that can be applied to a broad range of such problems, and may offer specific advantages for analysis in real-time computation and high-throughput processing.

We developed our analysis framework using a continuous and periodic stimulus applied to visual cortex cells. Implicit in our approach is the treatment of intrinsic imaging, as the signal dynamics can be easily described using our model. Also, with minor modifications, our framework can be easily extended to imaging protocols using other stimuli. For example, in some two-photon imaging experiments the stimulus is applied either in a random noise-like manner to avoid anticipatory responses [42], or by interspersing blank frames with no relevant excitatory or inhibitory stimulus present [9]. To apply our approach to data recorded from any of these experiments, we simply replace the stimulus represented as a harmonic regression in the current SCN model with an appropriate formulation of the stimulus model for the given protocol. The remainder of the analysis paradigm, including model fitting, model selection, goodness-of-fit assessment and inference, then proceeds as described above.

Our analytical framework is general so that a number of current statistical models that describe imaging modalities can be easily derived from it. For example, a commonly used model for fMRI data analysis [43] can be obtained from our Volterra series framework (see Methods) as
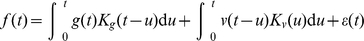


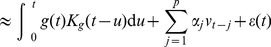
(5)


where 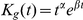 is a gamma function used to model the hemodynamic response of the body. The second term on the right represents physiological noise.

We assumed a signal plus Gaussian noise model in our analysis. However, in two-photon microscopy and other optical imaging modalities, the measured fluorescence intensity is a function of the discrete number of incident photons, and is therefore fundamentally a counting process and not necessarily Gaussian. The counting process nature of the two-photon experiments becomes more apparent as the acquisition rate increases [44]. The measured fluorescence in some two-photon imaging experiments may also exhibit non-Gaussian behavior due to distortions introduced during acquisition or post-processing. If the Gaussian assumption no longer holds, we can develop alternative likelihood approaches based on appropriately chosen non-Gaussian models. For example, neuronal spike trains can be extracted from two-photon data using template deconvolution [45], [46]. Non-Gaussian likelihoods based on the theory of point processes and implemented using the generalized linear model could be adapted to analyze these two-photon imaging data.

We modeled the time-series of neural responses in each pixel separately and did not consider inter-pixel dependencies. Such dependencies arise because: the activity of a single cell is captured across multiple pixels; retinotopy and network dependencies may lead to similar response in contiguous regions of the image; and data pre-processing procedures such as spatial smoothing introduce correlations. This problem, although challenging, currently confronts all biological imaging modalities and should ideally be studied by formulating appropriate biologically-based spatiotemporal models.

We have illustrated the application of our framework in offline analyses. However, due to its low computational complexity, our current analysis paradigm can be readily adapted to conduct large-volume, high-throughput imaging data analyses in real-time. These and other related aspects will be the topics of future reports.

## Methods

### Experimental procedures

Two-photon imaging of the fluorescent calcium indicator Oregon Green Bapta (OGB) was performed in the visual cortex of anesthetized ferrets. Neurons were bulk-loaded with OGB by intracortical injection of the AM-ester conjugated form of OGB using standard techniques [9], [18], [47]. Imaging was performed with a custom-made two-photon laser scanning microscope consisting of a modified Olympus Fluoview confocal scan head and a titanium/sapphire laser providing approximately 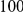 fsec pulses at 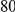 MHz pumped by a 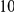 W solid-state source [48]. Fluorescence was detected using photomultiplier tubes in whole-field detection mode and a 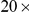, 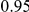 NA lens. Image acquisition was accomplished using Fluoview software. The images were taken from cortical layers 2/3, and this area was readily distinguished from layer 1 on the basis of the relative density of astrocytes and neurons. Visual stimuli, generated with Matlab using the PsychoPhysics Toolbox [49], were delivered via a 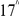 LCD display placed 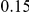 m away from the eyes of the animal. Neurons with relative fluorescence clearly distinguishable from the neuropil were chosen for subsequent cellular analysis.

### A Volterra series framework for signal plus colored noise imaging models

We assume that the measured fluorescence, 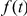, in a two-photon calcium imaging experiment is a function of a time-varying stimulus, 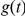, and noise in the system, 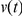. We further assume that the response, 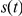, of the biological system depends on a nonlinear transformation of the stimulus input to the biological system. We can express the effect of the input stimulus and noise on the measured fluorescence at a pixel as
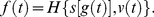
(6)


Expanding the right side of Eq. 6 in a Volterra series [50] yields
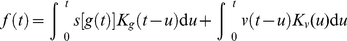


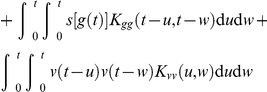


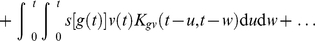
(7)


Take a discrete approximation to the first two terms on the right of Eq. 7 and assume that the second-order terms are sufficiently small so that they can be approximated as 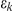, independent Gaussian noise with mean zero and variance 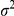. Then Eq. 7 becomes
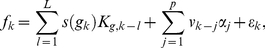
(8)


where 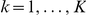 denotes the time-samples. We can express 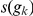 in terms of its Fourier series expansion. If 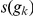 is periodic and smooth, its Fourier expansion can be well represented by a finite series. Thus, taking the first 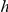 terms of the series, we can write
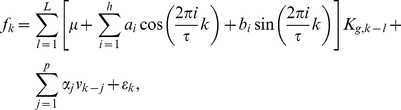
(9)


where 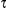 is the period of the input stimulus. In the two-photon imaging experiment, we assume that the effect of the stimulus on the system is instantaneous so that the discrete kernel can be written in terms of the Kronecker delta function as
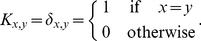
(10)


Substituting Eq. 10 into Eq. 9 yields
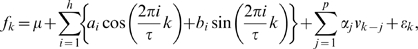
(11)


which is our model given in Eq. 1. If our model had not fit the data well, then we could use Eq. 7 to derive a modified model by including one or more of the second order terms.

### Burg algorithm

The Burg algorithm for autoregressive (AR) coefficient estimation uses least squares forward-backward prediction error minimization and is constrained to satisfy Levinson-Durbin recursions (LDR) [51], [52]. For the 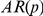 model in Eq. 3, the Burg algorithm estimates the coefficients 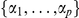 and innovations variance 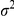, given the time series 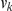 for 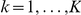, as follows [52].

**Require:** 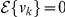 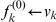 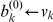 
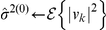


**for** 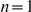 **to** 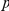 **do** 
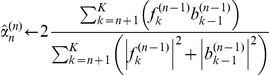
 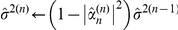


**if** 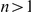 **then**

**for** 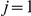 **to** 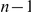 **do** 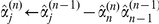


**end for**

**for** 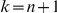 **to** 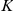 **do** 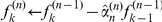


**end for**

**for** 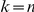 **to** 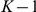 **do** 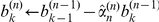


**end for**

**end for**

**end for** 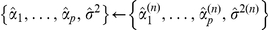


**return** 
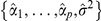


### Cholesky factorization

The 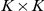 covariance matrix of the AR process 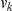 can be written in its Cholesky form as 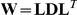. The inverse matrix 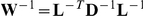 is used in our cyclic descent algorithm and can be calculated efficiently using Levinson-Durbin recursions, where [52]

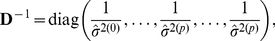
(12)


and
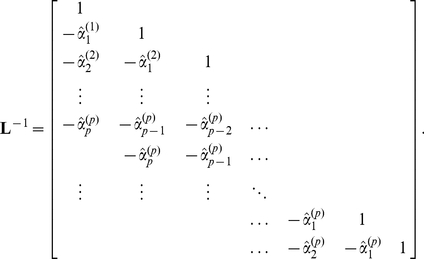
(13)


The coefficient and variance estimates of AR models up to order 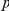 are computed by the Burg algorithm during 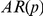 model parameter estimation and are therefore already available to populate 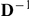 and 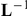. Hence this is a highly efficient procedure for computing 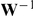 without matrix inversion.

### Cyclic descent algorithm

We use the cyclic descent algorithm for joint estimation of autoregressive and harmonic coefficient vectors, 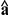 and 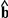, from the fluorescence data vector 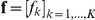. The algorithm proceeds as follows.
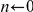


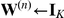


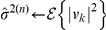



**repeat**

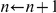


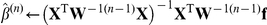


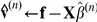



Compute 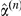 and 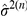 from 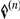 using Burg algorithm.

Compute 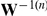 from its Cholesky factors using Levinson-Durbin recursion **untill** 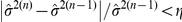


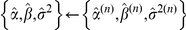



**return** 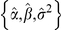


### Statistical methods

As our SCN model consists of two linear components, it is straight-forward to obtain confidence bounds and construct significance tests for the model parameters. For the 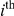 harmonic regression coefficient estimate, 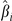, the approximate 95% confidence interval is 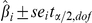 where 
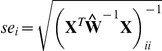
; 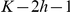; and 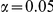 [53]. Similarly, for the 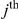 coefficient of the 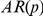 process, we have the confidence interval 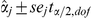 where 
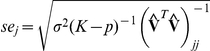
 and 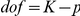. Here, 
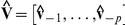
 contains the time-lagged samples of the 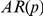 process and 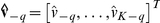. Based on these confidence intervals, we can design the t-test of significance for the model coefficients. The alternate hypothesis for the significance of the harmonic coefficients is 
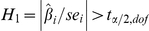
, and the alternative hypothesis for AR coefficient significance is defined similarly. The corresponding parameter is significant if the hypothesis is rejected.

We use the corrected Akaike information criterion (AICc) for model order selection. For the order 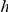 harmonic regression and 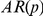 model, we define 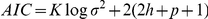 and
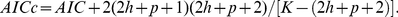
(14)


We use residual analysis to confirm the whiteness of our model's residual noise, 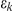. The normalized autocorrelation function (ACF) of the residuals at lag 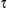 is given by 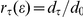, where 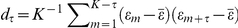. The approximate 95% whiteness bounds are 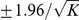 and the corresponding Ljung-Box portmanteau test statistic is 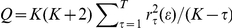, where conventionally 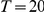 ACF taps are considered. The null hypothesis for the whiteness test is 
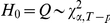
, where 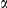 denotes the alpha level, taken as 5% in our analysis.

We use circular statistics to analyze circular random variables such as orientation 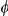. The circular mean is calculated as 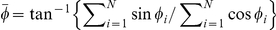 with its 95% confidence interval given by 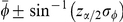, where 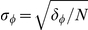 is circular standard error and
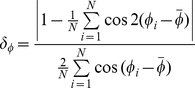
(15)


is circular dispersion [54].

### Ethics Statement

All experimental procedures were approved by the Massachusetts Institute of Technology Committee on Animal Care and adhered to the NIH guidelines for the Care and Use of Laboratory Animals (Protocol No. 0608-069-11).

## Supporting Information

Figure S1 **Two-photon fluorescence image of a cell population.** (a) Anatomical image of a population of 15 cells. Brighter gray shades represent higher fluorescence intensity. ROIs and cell indices indicate all of the cells identified manually. (b) Orientation tuning curve of each cell obtained by averaging the measured relative fluorescence across three trials.
(TIF)

Figure S2
**Convergence of the parameter estimates obtained with cyclic descent.** (a) Iterative estimates of the model parameters, namely the residual variance (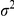), intercept (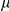), harmonic coefficients (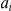 and 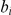; 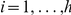), autoregressive coefficients (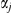; 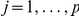) and residual variance (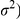, for the fluorescence time series in **Figure 3a**. (b) Percentage difference between the successive estimates of the model parameters in **a**. For the 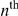 iteration, the percentage difference is calculated as 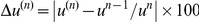, where 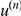 is the estimate of parameter 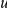 at the 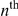 iteration and 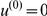. The y-axis has a logarithmic scale.(TIF)

Figure S3
**Model order selection.** (a) AICc surface for each of the cells in our data set averaged across the pixels for that cell (blue: low; red: high). (b) AICc as a function of the harmonic order when an AR component is not fit to the residual of the harmonic regression. (c) AICc as a function of the AR order when the optimal harmonic order from **b** is used. (d) Percentage of pixels of each cell that pass the Ljung-Box whiteness test (blue: 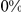; red: 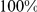).(TIF)

Table S1
**Optimal harmonic and AR model orders.** The table shows the optimal model orders for each cell, obtained using AICc and Ljung-Box test. Based on these results, we conclude that for our data set, a good fit to the data is obtained with approximately 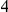 harmonics and 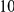 AR coefficients.(DOC)

## References

[1] Wilt B, Burns LD, Ho ETW, Ghosh KK, Mukamel EA (2009). Advances in light microscopy for neuroscience.. Annu Rev Neurosci.

[2] Denk W, Svoboda K (1997). Photon upmanship: why multiphoton imaging is more than a gimmick.. Neuron.

[3] Homma R, Baker BJ, Jin L, Garaschuk O, Konnerth A (2009). Dynamic Brain Imaging: Multi-Modal Methods and In Vivo Applications, Humana Pres., chapter Wide-field and two-photon imaging of brain activity with voltage- and calcium-sensitive dyes.

[4] Helmchen F, Denk W (2005). Deep tissue two-photon microscopy.. Nature Methods.

[5] Kerr JND, Denk W (2008). Imaging in vivo: watching the brain in action.. Nature Neuroscience.

[6] Majewska A, Sur M (2003). Motility of dendritic spines in visual cortex in vivo: Changes during the critical period and effects of visual deprivation.. Proc Natl Acad Sci.

[7] Barretto RPJ, Messerschmidt B, Schnitzer MJ (2009). In vivo uorescence imaging with highresolution microlenses.. Nature Methods.

[8] Holtmaat A, Bonhoeffer T, Chow DK, Chuckowree J, Paola VD (2009). Long-term, highresolution imaging in the mouse neocortex through a chronic cranial window.. Nat Protoc.

[9] Ohki K, Chung S, Ch'ng YH, Kara P, Reid CR (2005). Functional imaging with cellular resolution reveals precise micro-architecture in visual cortex.. Nature.

[10] Göbel W, Kampa BM, Helmchen F (2006). Imaging cellular network dynamics in three dimensions using fast 3D laser scanning.. Nature Methods.

[11] Kerr JND, Greenberg D, Helmchen F (2005). Imaging input and output of neocortical networks in vivo.. Proc Natl Acad Sci.

[12] Greenberg DS, Houweling AR, Kerr JND (2008). Population imaging of ongoing neuronal activity in the visual cortex of awake rats.. Nature Neuroscience.

[13] Yaksi E, Friedrich RW (2006). Reconstruction of firing rate changes across neuronal populations by temporally deconvolved Ca^2+^ imaging.. Nature Methods.

[14] Mukamel EA, Nimmerjahn A, Schnitzer MJ (2009). Automated analysis of cellular signals from large-scale calcium imaging data.. Neuron.

[15] Garaschuk O, Linn J, Eilers J, Konnerth A (2000). Large-scale oscillatory calcium waves in the immature cortex.. Nature.

[16] Mironov SL (2008). Metabotropic glutamate receptors activate dendritic calcium waves and TRPM channels which drive rhythmic respiratory patterns in mice.. J Physiol.

[17] Basole A, White LE, Fitzpatrick D (2003). Mapping multiple features in the population response of visual cortex.. Nature.

[18] Schummers J, Yu H, Sur M (2008). Tuned responses of astrocytes and their inuence on hemodynamic signals in the visual cortex.. Science.

[19] Martinez LM, Alonso JM, Reid CR, Hirsch JA (2002). Laminar processing of stimulus orientation in cat visual cortex.. J Physiol.

[20] Gabbay M, Brennan C, Kaplan E, Sirovich L (2000). A principal components-based method for the detection of neuronal activity maps: application to optical imaging.. NeuroImage.

[21] Kalatsky VA, Stryker MP (2003). New paradigm for optical imaging: temporally encoded maps of intrinsic signal.. Neuron.

[22] Mrsic-Flogel T, Hübener M, Bonhoeffer T (2003). Brain mapping: New wave optical imaging.. Curr Biol.

[23] Sornborger A, Sailstad C, Kaplan E, Sirovich L (2003). Spatiotemporal analysis of optical imaging data.. NeuroImage.

[24] Purdon PL, Solo V, Weisskoff RM, Brown EN (2001). Locally regularized spatiotemporal modeling and model comparison for functional MRI.. NeuroImage.

[25] Kantz H, Schreiber T (2004). Nonlinear Time Series Analysis..

[26] Kantz H, Schreiber T (1993). Nonlinear noise reduction: A case study on experimental data.. Phys Rev E.

[27] Schreiber T, Grassberger (1991). A simple noise-reduction method for real data.. Phys Lett A.

[28] Effern A, Lehnertz K, Schreiber T, Grunwald T, David P (2000). Nonlinear denoising of transient signals with application to event-related potentials.. Physica D.

[29] Corradi C (1979). A note on the computation of maximum likelihood estimates in linear regression models with autocorrelated errors.. J Econometrics.

[30] Perc M, Green AK, Marhl M (2008). Establishing the stochastic nature of intracellular calcium oscillations from experimental data.. Biophys Chem.

[31] Perc M, Rupnik M, Gosak M, Marhl M (2009). Prevalence of stochasticity in experimentally observed responses of pancreatic acinar cells to acetylcholine.. Chaos.

[32] Marhl M, Gosak M, Perc M, Roux E (2010). Importance of cell variability for calcium signaling in rat airway myocytes.. Biophys Chem.

[33] Bullmore E, Long C, Suckling J, Fadili J, Calvert G (2001). Colored noise and computational inference in neurophysiological (fMRI) time series analysis: Resampling methods in time and wavelet domains.. Human Brain Mapping.

[34] Chen Y, Geisler WS, Seidemann E (2008). Optimal temporal decoding of neural population responses in a reaction-time visual detection task.. J Neurophysiol.

[35] Brown EN, Choe Y, Luithardt H, Czeisler CA (2000). A statistical model of the human coretemperature circadian rhythm.. Am J Physiol Endocrinol Metab.

[36] Brunel N, Latham PE (2003). Firing rate of the noisy quadratic integrate-and-fire neuron.. Neural Comput.

[37] Destexhe A, Rudolph M, Fellous JM, Sejnowski TJ (2001). Fluctuating synaptic conductances recreate in vivo-like activity in neocortical neurons.. Neuroscience.

[38] Sakai Y, Funahashi S, Shinomoto S (1999). Temporally correlated inputs to leaky integrate-and-fire models can reproduce spiking statistics of cortical neurons.. Neural Networks.

[39] Huang MC, Wu JW, Luo YP, Petrosyan KG (2010). Fluctuations in gene regulatory networks as Gaussian colored noise.. J Chem Phys.

[40] Gibson JD, Koo B, Gray SD (1991). Filtering of colored noise for speech enhancement and coding.. IEEE Trans Sig Proc.

[41] Shenderov AD, Sheetz MP (1997). Inversely correlated cycles in speed and turning in an ameba: An oscillatory model of cell locomotion.. Biophys J.

[42] Everson RM, Prashanth AK, Gabbay M, Knight BW, Sirovich L (1998). Representation of spatial frequency and orientation in the visual cortex.. Proc Natl Acad Sci.

[43] Friston KJ, Josephs O, Rees G, Turner R (1998). Nonlinear event-related responses in fMRI.. Magn Reson Med.

[44] Sjulson L, Miesenböck G (2007). Optical recording of action potentials and other discrete physiological events: a perspective from signal detection theory.. Physiology.

[45] Vogelstein JT, Watson BO, Packer AM, Yuste R, Jedynak B (2009). Spike inference from calcium imaging using sequential Monte Carlo methods.. Biophys J.

[46] Ozden I, Lee HM, Sullivan MR, Wang SH (2008). Identification and clustering of event patterns from in vivo multiphoton optical recordings of neuronal ensembles.. J Neurophysiol.

[47] Stosiek C, Garaschuk O, Holthoff K, Konnerth (2003). In vivo two-photon calcium imaging of neuronal networks.. Proc Natl Acad Sci.

[48] Majewska A, Yiu G, Yuste R (2000). A custom-made two-photon microscope and deconvolution system.. Eur J Physiol.

[49] Brainard DH (1997). The Psychophysics Toolbox.. Spatial Vision.

[50] Schetzen M (2006). The Volterra and Wiener Theories of Nonlinear Systems..

[51] Box GEP, Jenkins GM, Reinsel GC (2008). Time Series Analysis..

[52] Kay SM (1999). Modern Spectral Estimation..

[53] Myers RH, Montgomery DC, Vining GG (2002). Generalized Linear Models..

[54] Fisher NI (1993). Statistical Analysis of Circular Data..

