# Supplementary figures and images for: Denoising Two-Photon Calcium Imaging Data
Source: PLoS One. 2011 Jun 7;6(6):e20490. doi: 10.1371/journal.pone.0020490 (PMC3110192; doi:10.1371/journal.pone.0020490)

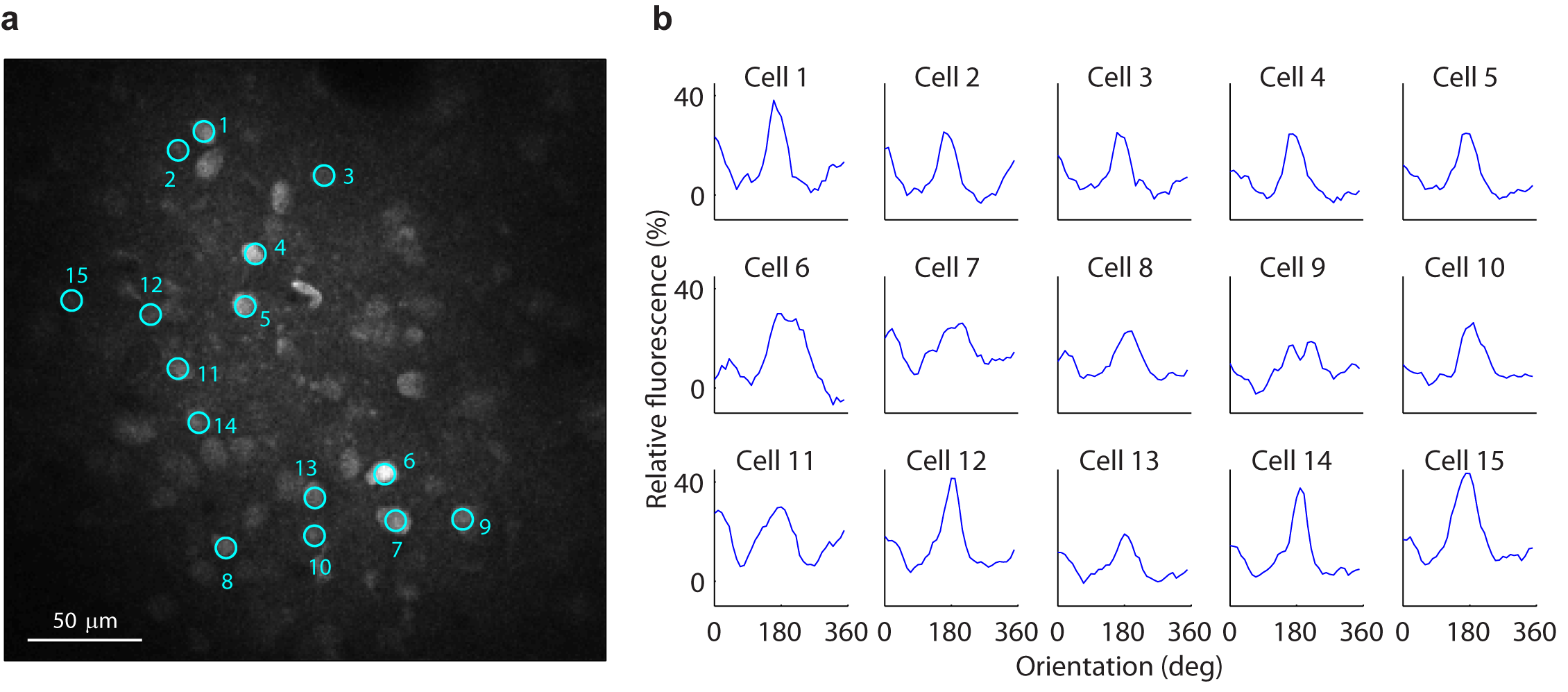

Supplement: Figure S1 — Two-photon fluorescence image of a cell population. (a) Anatomical image of a population of 15 cells. Brighter gray shades represent higher fluorescence intensity. ROIs and cell indices indicate all of the cells identified manually. (b) Orientation tuning curve of each cell obtained by averaging the measured relative fluorescence across three trials. (TIF) [file pone.0020490.s001.tif]

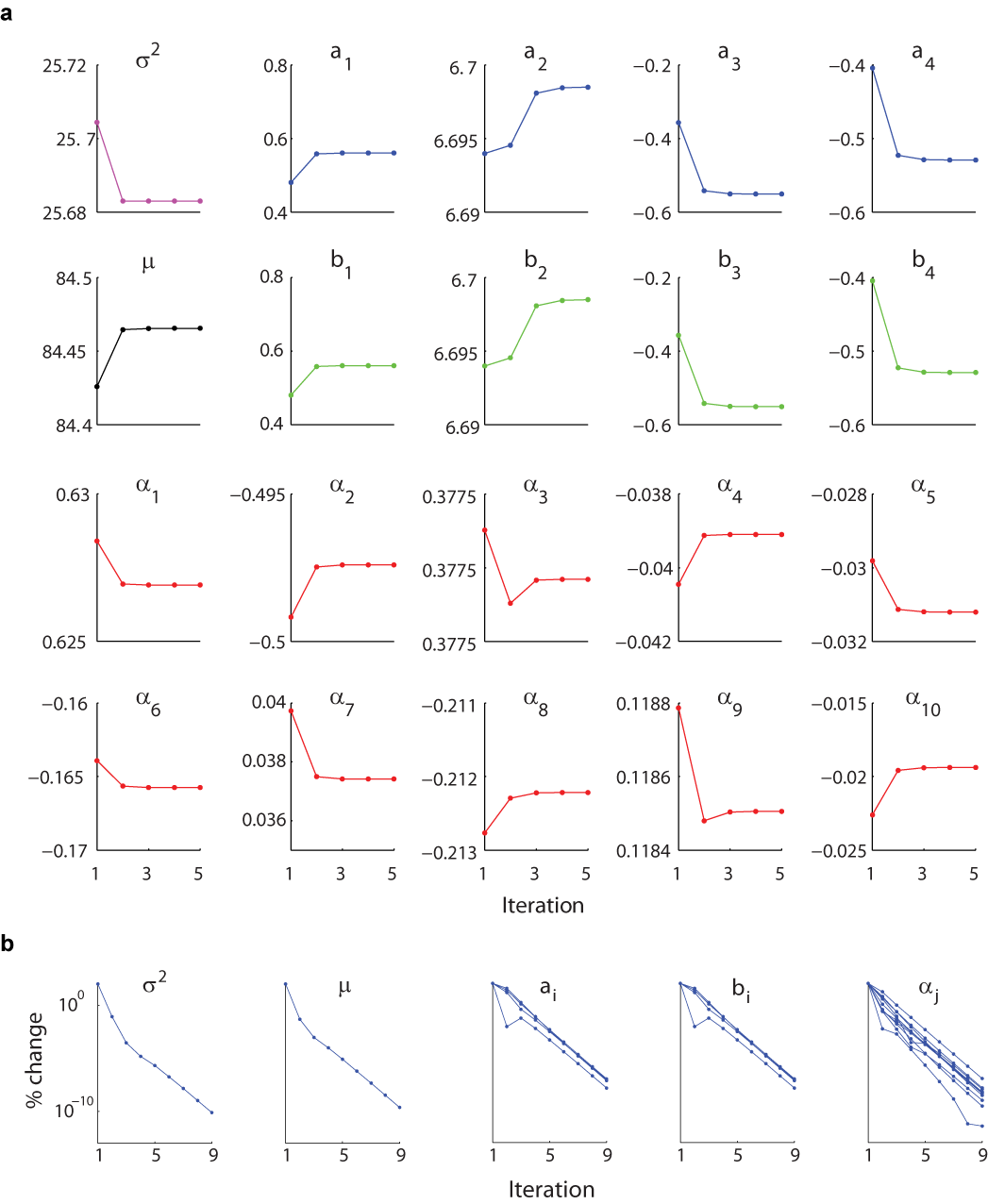

Supplement: Figure S2 — Convergence of the parameter estimates obtained with cyclic descent. (a) Iterative estimates of the model parameters, namely the residual variance (), intercept (), harmonic coefficients ( and ; ), autoregressive coefficients (; ) and residual variance (, for the fluorescence time series in Figure 3a . (b) Percentage difference between the successive estimates of the model parameters in a. For the iteration, the percentage difference is calculated as , where is the estimate of parameter at the iteration and . The y-axis has a logarithmic scale. (TIF) [file pone.0020490.s002.tif]

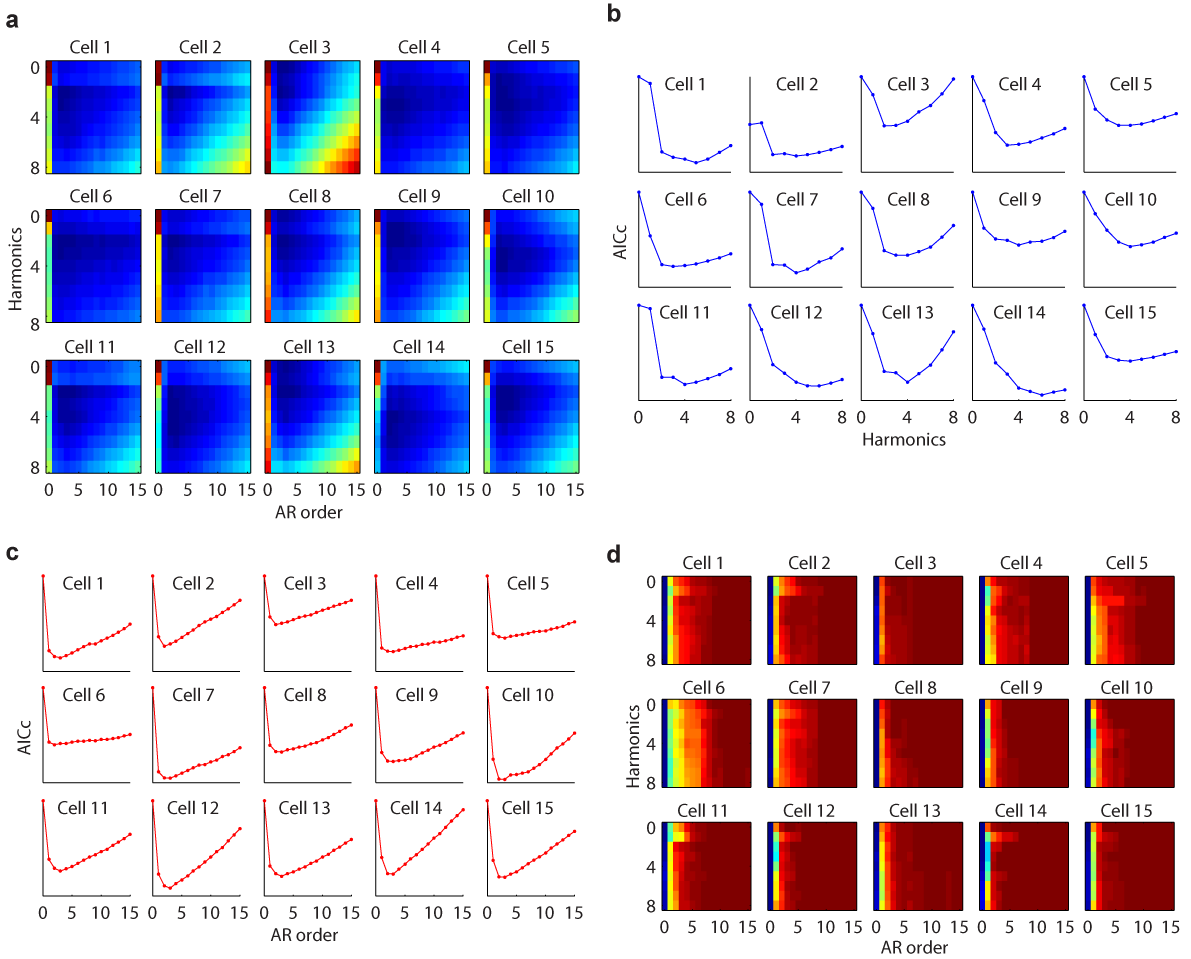

Supplement: Figure S3 — Model order selection. (a) AICc surface for each of the cells in our data set averaged across the pixels for that cell (blue: low; red: high). (b) AICc as a function of the harmonic order when an AR component is not fit to the residual of the harmonic regression. (c) AICc as a function of the AR order when the optimal harmonic order from b is used. (d) Percentage of pixels of each cell that pass the Ljung-Box whiteness test (blue: ; red: ). (TIF) [file pone.0020490.s003.tif]
