# Supplementary material for: Denoising Two-Photon Calcium Imaging Data
Source: PLoS One. 2011 Jun 7;6(6):e20490. doi: 10.1371/journal.pone.0020490 (PMC3110192; doi:10.1371/journal.pone.0020490)
Supplement: Table S1 — Optimal harmonic and AR model orders. The table shows the optimal model orders for each cell, obtained using AICc and Ljung-Box test. Based on these results, we conclude that for our data set, a good fit to the data is obtained with approximately harmonics and AR coefficients. (DOC) [file pone.0020490.s004.doc]

Table S1

| Cell index | Optimal harmonic order from AICc | Optimal AR order from AICc | Optimal AR order from LB criterion |
| --- | --- | --- | --- |
| 1 | 5 | 3 | 10 |
| 2 | 4 | 2 | 9 |
| 3 | 2 | 2 | 7 |
| 4 | 3 | 3 | 9 |
| 5 | 3 | 3 | 11 |
| 6 | 3 | 2 | 11 |
| 7 | 4 | 3 | 9 |
| 8 | 3 | 3 | 6 |
| 9 | 4 | 3 | 10 |
| 10 | 4 | 3 | 8 |
| 11 | 4 | 3 | 8 |
| 12 | 6 | 3 | 6 |
| 13 | 4 | 3 | 7 |
| 14 | 6 | 3 | 8 |
| 15 | 4 | 3 | 4 |
